# Transcriptome profiling reveals the genes and pathways involved in thermo-tolerance in wheat *(Triticum aestivum* L.) genotype Raj 3765

**DOI:** 10.1038/s41598-022-18625-7

**Published:** 2022-09-01

**Authors:** Mawuli K. Azameti, Alok Ranjan, P. K. Singh, Kishor Gaikwad, Anil Kumar Singh, Monika Dalal, Ajay Arora, Vandna Rai, Jasdeep C. Padaria

**Affiliations:** 1 PG School, ICAR-Indian Agricultural Research Institute, New Delhi, 110012 India; 2 ICAR-National Institute for Plant Biotechnology, New Delhi, 110012 India; 3 Division of Genetics, Indian Agricultural Research Institute, Pusa, New Delhi, 110012 India; 4 Division of Plant Physiology, Indian Agricultural Research Institute, Pusa, New Delhi, 110012 India; 5 Present Address: CSIR-Food Research Institute, Accra, Ghana

**Keywords:** Biotechnology, Molecular biology, Plant sciences

## Abstract

Wheat, one of the most widely consumed staple food crops globally, is relatively vulnerable to high temperature-induced heat stress. It is therefore essential to gain more insight into the comprehensive mechanism of thermotolerance of wheat in order to safeguard its production. In view of this, we analysed heat stress responsive transcriptome data of wheat to determine its gene expression level under heat stress. A total of 7990 DEGs, including 4483 up-regulated and 3507 down regulated genes were identified. Gene Ontology (GO) analysis categorized 3910 DEGs into different ontology families. 146 pathways involving 814 DEGs were enriched during KEGG analysis. Metabolic pathways and biosynthesis of secondary metabolites were the major pathways enriched. MYB (myeloblastosis) transcription factors (TFs) and many other TFs as bHLH, WRKY, NAC, ERF, were determined to be quite abundant in the DEGs. Since various reports indicate that these TFs play important role in plants abiotic stress, it is an indication that our DEGs are functional in heat stress tolerance. Verification of few selected DEGs using RT-qPCR produced expression levels similar to the transcriptome data. This indicates that the transcriptome data is reliable. These results could be helpful in enhancing our understanding of the mechanism underlying thermotolerance in wheat.

## Introduction

Various climatic parameters continue to change, such as rise in global average atmospheric temperature. The global average temperature is expected to increase with its accompanied water stress as a result of decline in rainfall^1^. Lorenz et al.^2^ reported that 66.7% of the warming has occurred since 1975 at a rate of 0.15–0.20 °C per decade, while Hansen et al.^3^ puts the rate at 0.18 °C every decade. Additional 198 million tonnes of wheat would be needed worldwide by 2050 to meet the future demands of food, for which 77% increase in wheat production would be needed in developing countries^4^.

Wheat is globally regarded as one of the most widely cultivated crop plants which form a major aspect of basic human nutrition in many areas. It is a source of many important nutrients such as vitamins, and starch^5^. Wheat, being a cool season crop, grows best at a day time temperature of 15 °C at the reproductive stage. A 3–4% reduction in yield has been observed for each degree Celsius above the optimum temperature^6^. Globally, the average temperature is increasing at a rate of 0.18 °C per decade^4^. Consequently, the combination of warm climate and extreme elevated temperature have increased the detrimental pressure on wheat production^7^. As the ambient temperature increases, the global wheat grain production becomes negatively affected^8^. High temperature increases the rate of photorespiration in wheat. It also inhibits photosynthesis^9^, and increases early senescence, which is associated with smaller plants^8^. These pose a serious danger to the world food security.

To address the increasing demand for wheat grain and also to safeguard its production, it is imperative to understand the molecular mechanisms underlying thermo-tolerance and subsequently develop wheat cultivars resistant to heat stress.

Researchers worldwide have used transcriptome sequencing to identify differentially expressed genes (DEGs) and analyse transcriptome changes of genes under various stress conditions^10–12^. Few studies were conducted on transcriptome profiling of wheat under heat stress^8,13,14^. However, there is still the need to further unravel the roles of genes involved in heat signalling cascades or in the conversion of metabolites.

In this study, we analysed available heat responsive transcriptome sequencing data of wheat genotype Raj 3765 to unravel the molecular mechanism of wheat response to heat and to identify candidate genes playing roles in wheat thermotolerance.

## Materials and methods

### Plant materials and heat treatments

Seeds of a panel of sixteen (16) genotypes contrasting for thermotolerance used in this study were obtained from Dr P.K. Singh, Principal Scientist, Division of Genetics, Indian Agricultural Research Institute (affiliated institute), New Delhi, India. The seeds of the popularly grown genotypes of wheat are maintained as per institutional guidelines. All procedures performed in the study were in compliance with relevant institutional, and national regulations. The seeds of the genotypes were pre-vernalized, germinated in petri plates, and later transferred to twelve inches pots which contained soilrite and grown under greenhouse conditions at the National Phytotron Facility, New Delhi. The temperature was set at 24 ± 2 °C, light intensity at 350 μmol/m/s, with 16/8 h photoperiod and 60% humidity. The plants were watered once a day. At the post-anthesis stage (Feekes scale-10.53), the plants were subjected to heat stress (HS) at 42 °C for six (6) continuous hours in an incubator chamber by increasing the temperature at 1 °C per 10 min until the desired temperature was attained. Leaf samples were then after collected from three biological replicates. The temperature of the control plants was kept at (24 ± 2 °C). The leaf samples collected were immediately frozen in liquid nitrogen and stored at − 80 °C for further molecular biology experimentation.

### RNA-seq data processing and transcriptome assembly

An available heat stress-responsive transcriptome data of wheat genotype Raj 3765 generated in our previous experiment, and submitted to the SRA database with SRA IDs; SRR16347581, and SRR16347579 for control and treated (42 °C for 6 h) samples respectively was analysed and used for the gene expression studies. The Raj 3765 plants at the post-anthesis stage were exposed to heat stress at 42 °C for six (6) hours. The flag leaves were collected to generate the heat stress responsive transcriptomic data.

Quality of the raw reads were checked using FastQC^15^. After processing, high-quality filtered reads were combined for de novo assembly using Trinity (vr2012-05-18) tool (http://trinityrnaseq.sourceforge.net) at default parameters. Parameters like k-mer length (default k-mers i.e. 25), expected coverage, and insert length were optimized to obtain good assembly.

High-quality reads were clustered by CD-HIT V4.6 (version 4.5.4 4) to remove redundancies and unigenes were obtained with sequence identity^16^.

### Identification of differentially expressed heat-responsive genes (DEGs)

Different libraries containing differentially expressed genes (DEGs) were analysed using the FPKM method and the DEGs identified with edgeR package^17^. A number of mapping reads for each unigene was determined by FPKM, and the unigene expression levels were assessed. Determination of the normalization factors was carried out by the use of the trimmed mean of M-values method and p values were calculated using negative binomial distribution methods. Multiple tests were adjusted using Benjamini–Hochberg methods. Significant differentially expressed unigenes were determined using the false discovery rate (FDR) < 0.001 and a p-value < 0.05. Unigenes having length < 200 bp and FPKM < 1 were eliminated to avoid any possible assembly errors and to ensure the quality of the resulted assembly. Unigenes with high quality were used in further analysis. High quality reads were used to map back to the assembled transcripts. The significant DEGs (p ≤ 0.05 and log2F) were identified for further studies.

### Functional annotation and classifications

Functional annotation was done through a BLASTx search with an *E-*value cut-off of ≤ 1e−5 against the non-redundant (NR) protein database of NCBI (E value < 1.0E−5). Gene ontology determination of the up and down-regulated genes was carried out using Blast2GO version 2.8 (https://www.blast2go.com/). KEGG mapping was carried out using Bast2GO tool for analysis of biochemical pathways of annotated unigenes. Both the pathway enrichment analyses and GO was performed at P-value with a cut-off of 0.05.

### Identification of transcription factors (TFs)

The transcripts obtained were searched against transcription factor protein sequences present in the nr database.

### In silico mining of simple sequence repeats (SSRs) from transcriptome data

The identification of SSRs in *Raj* 3765 genotype was carried out using MISA (MIcroSAtellite) software tool, a Perl script-based software tool with default parameters^18^. Further, the presence of SSR motifs in the coding or untranslated region (UTR) of the gene was determined by examining the aligned portions of sequences with matches to annotated protein-coding orthologs.

### Extraction of RNA and gene expression validation by real time-quantitative polymerase chain reaction (RT-qPCR)

Eight (8) DEGs (four up-regulated and four down-regulated) were selected for validation using RT-qPCR. Table 1 contains the description of the genes and their specific primers. TRIzol Reagent was used in isolating the total RNA according to standard protocol. The quality of the total RNA was determined using electrophoresis while the concentration was determined by optical density at A260/A280 using NanoDrop (Thermo Fisher Scientific, USA). The RNA was treated with DNase (Sigma-Aldrich, USA) to remove any trace of DNA contamination. cDNA was synthesized from the RNA isolated using Superscript III first strand cDNA synthesis system (Invitrogen, USA). Primers were designed using the available EST sequence and Primer3 software. Primers of 5OD and 25 nm were synthesised from the Integrated DNA Technologies Inc. (IDT, USA). Each PCR reaction (20 μl) was made up of 10 μl of Lightcycler 480 SYBR green Master mix (Roche, Germany), 1 μl of cDNA (100 ng), 0.5 μl each of forward and reverse primers (10 pmol), and 8 μl of nuclease free water. Amplification was done using the following program; 95 °C for 3 min, followed by 40 cycles (95 °C for 10 s, 60 °C for 10 s, 72 °C for 10 s). The internal control used was *β*-actin gene, with accession no. AB181991.1. Each reaction was carried out in three replicates. 2^−ΔΔCt^ method was used to determine the relative fold change values between the experimental and calibrator samples^19^. Melting curve analysis was used to monitor the primer-template specificity.

**Table 1:** List of the genes and specific primers used in the validation using RT-qPCR.

No.	Gene name	Primer sequences (5′–3′)
1	HSP 90.1-B1	F-CGTGTCCAGTCGAAGTTAGTC R-ACATCGCCAGAAGACACATAG
2	HSP 101b-A	F-CTGAAGTGCCTGTCGGATAAA R-ACACGCGTCACAGAACAA
3	HSP 101c-B	F-GGGAAGGTGATACTGTTCATCG R-ATCGGCTTGAACAGGTTGG
4	*Triticum aestivum* mRNA clone	F-GTCTCTGGAACTCCTGCAAT R-CGTAGGGACTTCGGAAATGT
5	*Aegilops tauschii* uncharacterized mRNA	F-GAGATGTCAACATGGCAAAGG R-CGAGTTGTAACCACAAGTGAAA
6	*Aegilops tauschii* uncharacterized transcript variant X3 mRNA	F-CTATCCGTATCAGTGGGCTATG R-CTATTCCCTCTGGCTCTTCTG
7	*Aegilops tauschii* uncharacterized transcript variant X5 mRNA	F-CATCGAGGCCAAGGTGAA R-GCCCTGCCAGATCCAAT
8	*Aegilops tauschii* elongation factor 2-like mRNA	F-CACTTGGTGGCATCTATGGT R-GGTAGGCCTTGATGTTGTAGAG

## Results

### Data analysis and identification of differentially expressed genes (DEGs) in response to heat treatment

**Figure 1:**
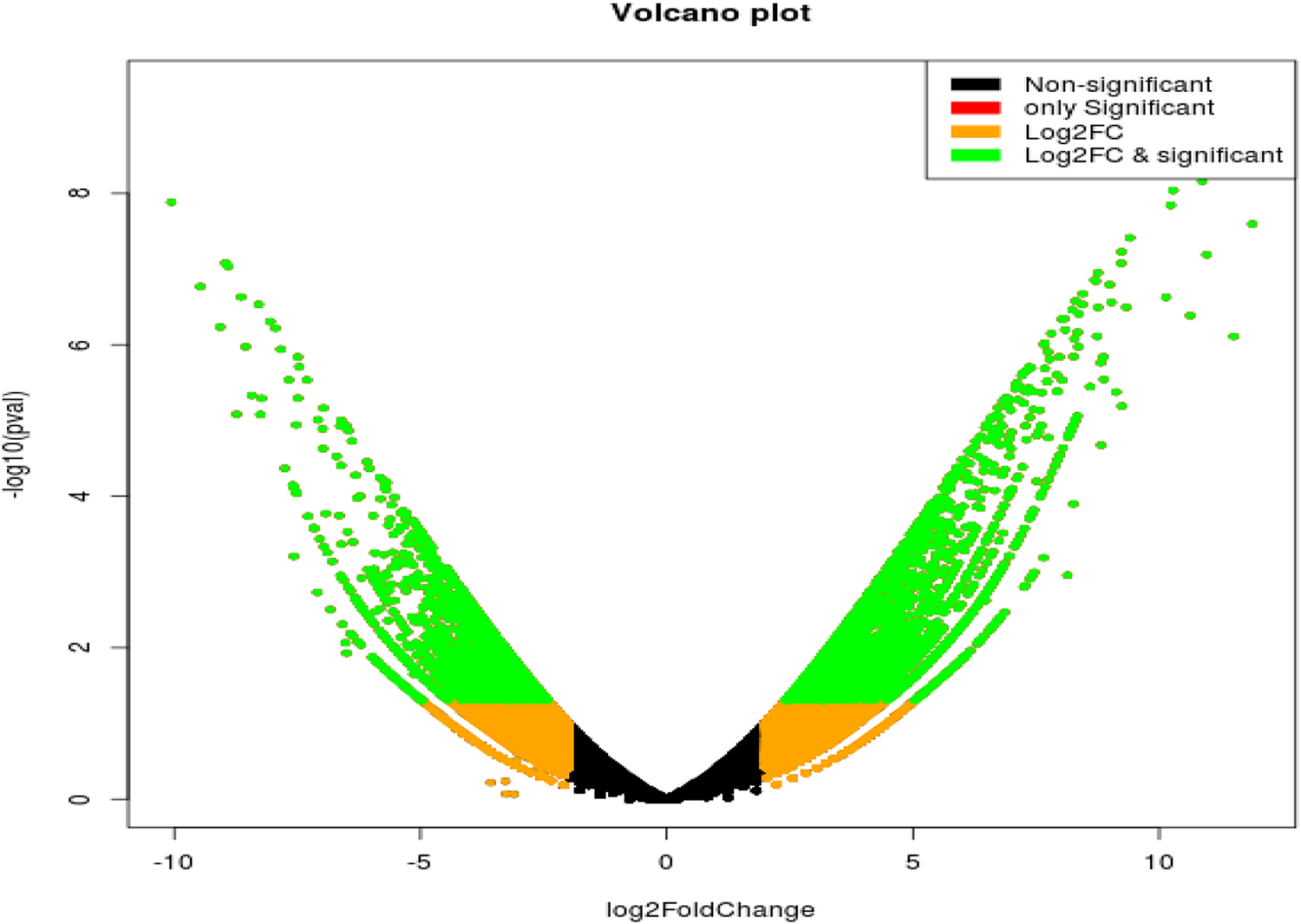
Analysis of differentially expressed genes (DEGs) in wheat flag leaf represented by volcano plot.

**Figure 2:**
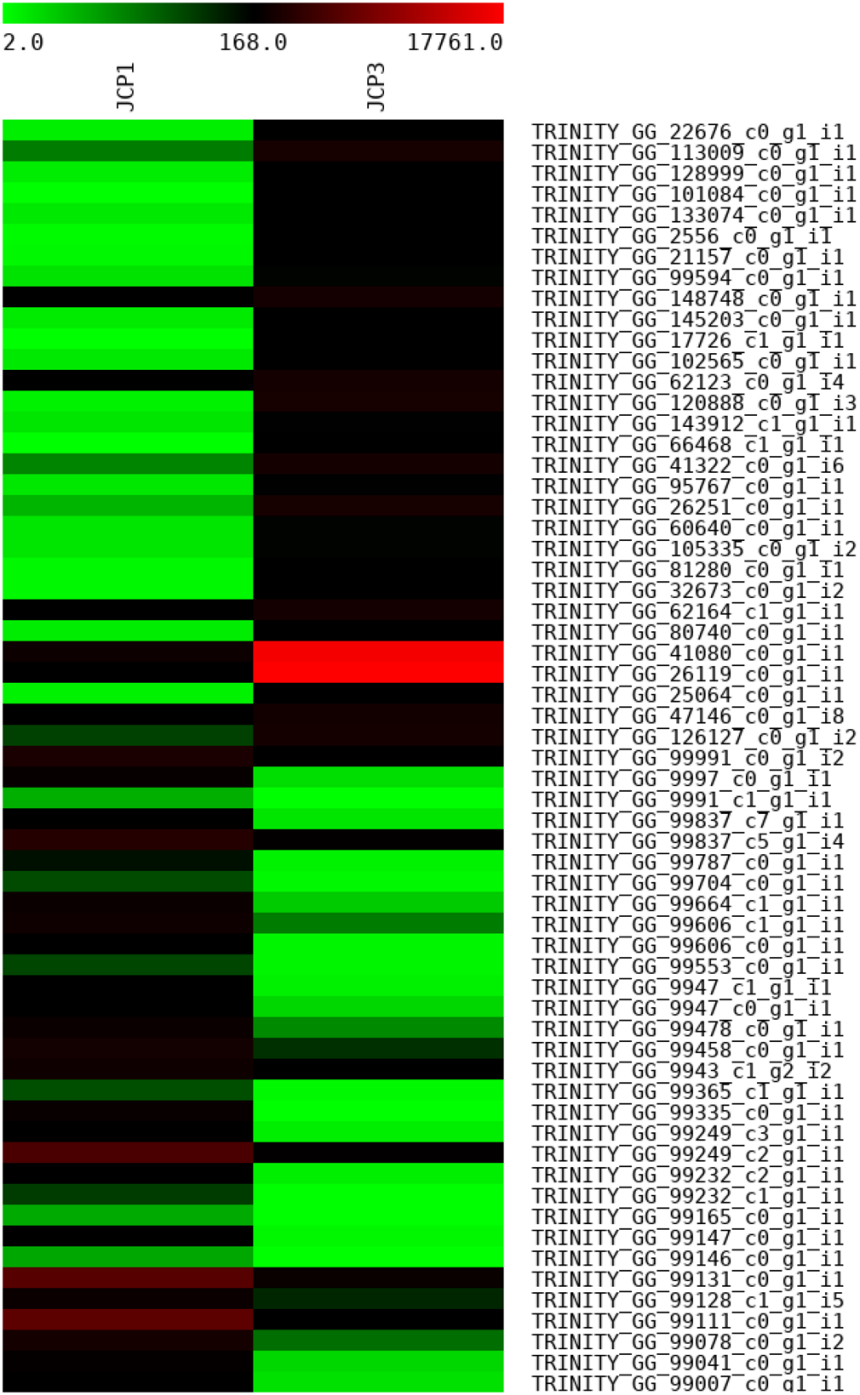
Heatmap analysis of the up and down regulated DEGs between the control and the heat-stress treated wheat genotype Raj 3765. The upper 30 genes are the up-regulated DEGs while the lower 30 genes are the down-regulated DEGs.

RNA-seq data from wheat genotype Raj 3765 flag leaf exposed to high temperature (42 °C) for 6 h (SRR16347581, and SRR16347579) were analysed. A total of 237,586 trinity transcripts were generated with GC percentage of 47.53. A total of 7990 significant DEGs, comprising 4483 up-regulated and 3507 down-regulated genes, to heat stress were identified. Volcano plots were employed in the visualization of the number of transcripts that were significantly regulated during heat treatment (Fig. 1). The negative values indicate down regulation while the positive values represent the up regulation. The significantly up and down regulated DEGs are shown in green dots according to the criteria of |log2FC| ≥ 2 and p-values (p < 0.05). The results indicated that there were more upregulated genes than the downregulated genes in flag leaf of wheat genotype Raj 3765 after 6 h of heat treatment. Heatmap analysis of the first 30 up and down regulated DEGs is represented in Fig. 2.

### Gene ontology classification of differentially expressed genes

**Figure 3:**
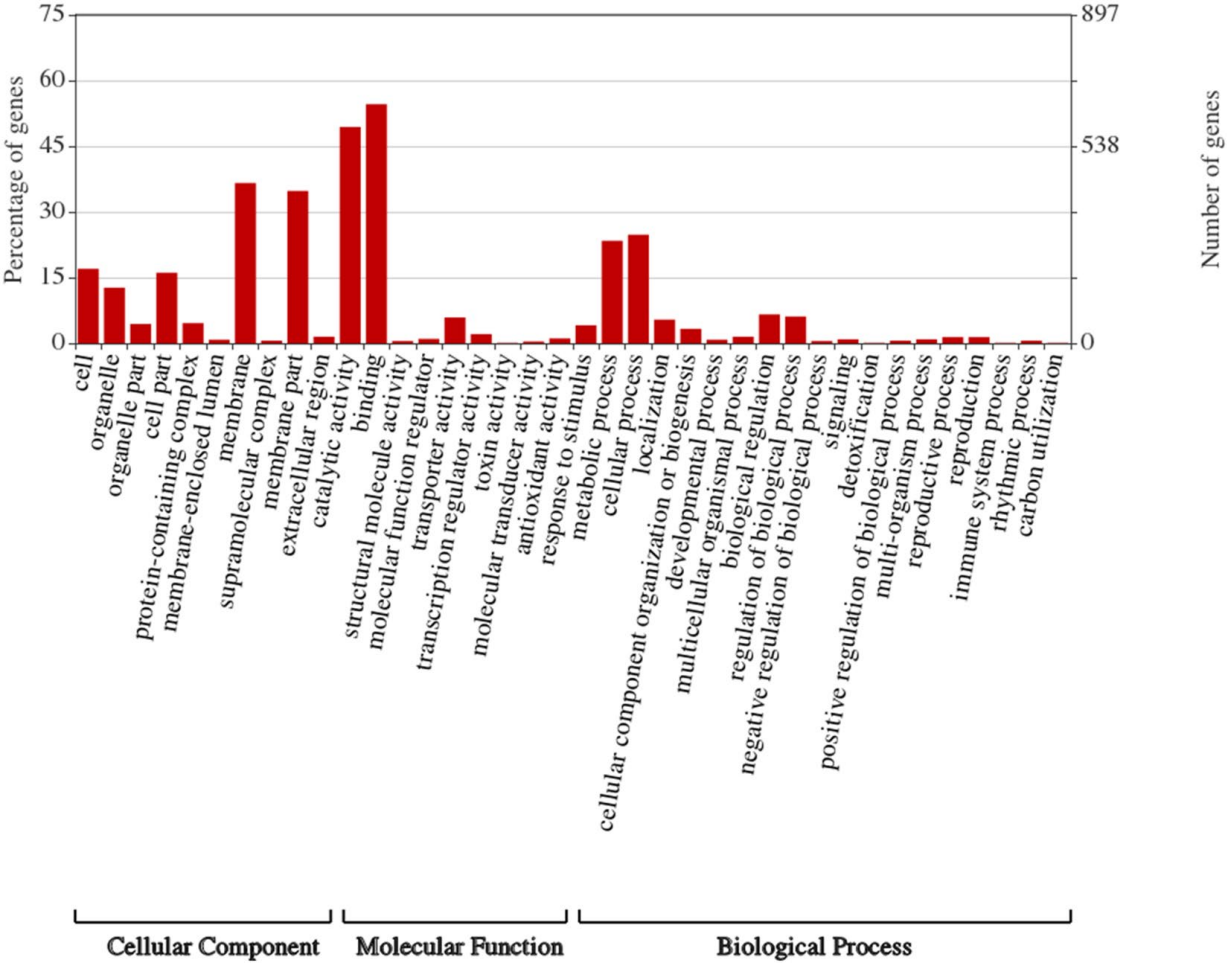
Gene ontology classification analysis of the DEGs between control and heat-stressed flag leaves of wheat genotype Raj 3765. GO functions were represented in X-axis; the number of DEGs annotated in each GO term was presented in left Y-axis; and the right Y-axis showed the percentage of DEGs which were annotated in each GO term.

GO term enrichment analysis was done to determine specific molecular factors involved in thermotolerance in wheat flag leaf DEGs (Fig. 3).

**Figure 4:**
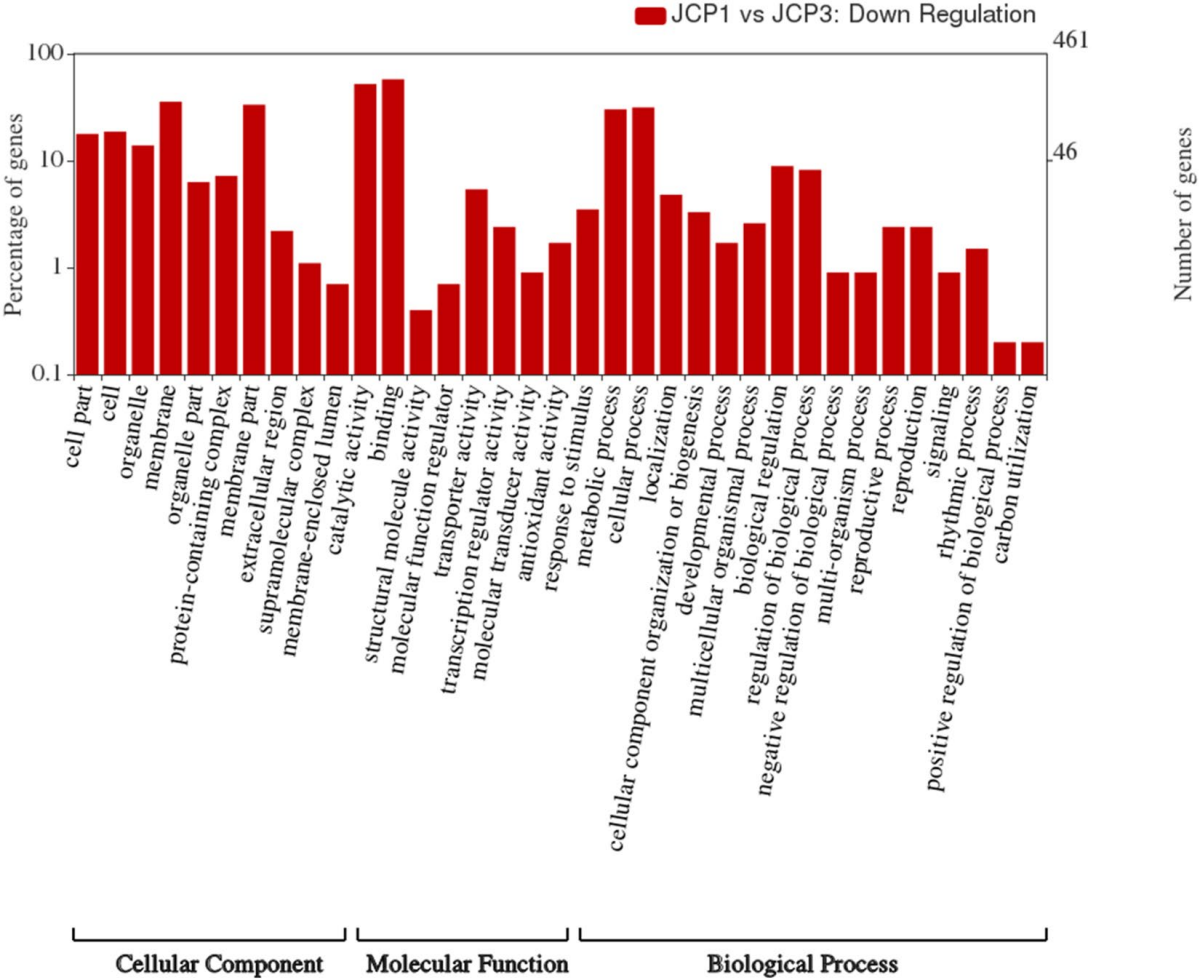
Gene ontology classification analysis of the down-regulated DEGs. GO functions were represented in X-axis; the number of DEGs annotated in each GO term was presented in left Y-axis; and the right Y-axis showed the percentage of DEGs which were annotated in each GO term.

**Figure 5:**
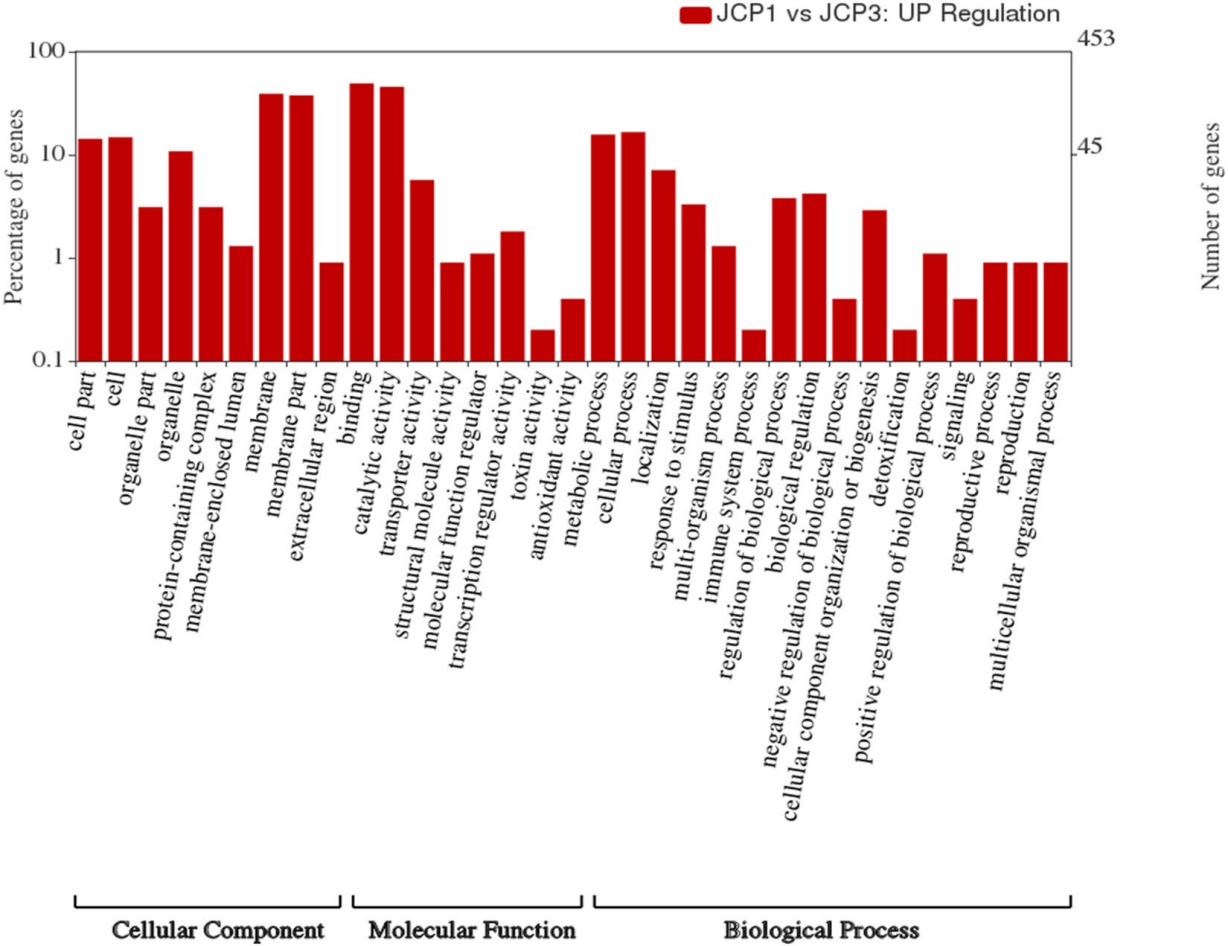
Gene ontology classification analysis of the up-regulated DEGs. GO functions were represented in X-axis; the number of DEGs annotated in each GO term was presented in left Y-axis; and the right Y-axis showed the percentage of DEGs which were annotated in each GO term.

The ontology analysis was carried out to determine three sub parts: biological process (BP), molecular function (MF), and cellular component (CC). Our analysis of the DEGs showed that 48.93% (3910) of the DEGs were functionally categorized into different ontology families. Out of this, 988 were found to be playing roles in biological processes, 1545 for cellular component and 1377 genes performing molecular functions. We also carried out the gene ontology analysis for the up and down regulated genes separately (Figs. 4 and 5).

Analysis of the up-regulated genes showed that 271 genes were involved in biological processes, while 567 were for cellular component, and 476 for molecular functions. The results of the analysis revealed that proteins that are encoded by the up-regulated genes were significantly associated to 16 biological processes (BPs). Majority of the up-regulated genes belong to GO:0009987 (cellular process) and GO:0008152 (metabolic process).

The cellular component ontology analysis of the up-regulated genes revealed that majority of the gene counts were annotated with the GO:0016020 (membrane), GO:0044425 (membrane part), GO:0005623 (cell), and GO:0044464 (cell part) (Fig. 4). Majority of the down-regulated genes were annotated to GO:0009506 (plasmodesma), GO:0005730 (nucleolus), GO:0022625 (cytosolic large ribosomal subunit), and GO:0046658 (anchored component of plasma membrane).

Molecular function terms give a description of the activities the gene products perform at the molecular level. Majority of the up-regulated genes were annotated to GO:0005488 (binding) and GO:0003824 (catalytic activity).

479 down-regulated genes were associated with biological processes, while 630 and 559 were associated with cellular component, and molecular functions respectively (Fig. 5). Result shows that majority of the genes involved in the biological processes were concerned with cellular processes (GO:0009987), and metabolic processes (GO:0008152). Within molecular functions category, GO:0005488 (binding), and catalytic activity (GO:0003824) were mostly overrepresented. In the cellular component category, the down-regulated genes were mostly associated with the membrane (GO:0016020), membrane part (GO:0044425), cell (GO:0005623), and cell part (GO:0044464).

In addition, GO enrichment analysis was performed to gain insight into the molecular functions of DEGs in response to heat stress. The GO molecular terms like, transcription factor activity (GO:0003700), unfolded protein binding (G0:0051082), protein self-association (GO:0043621), heat shock protein binding (GO:0031072), sequence-specific DNA binding (GO:0043565) and ATP binding (GO:0005524) were highly enriched among DEGs in response to heat stress (Supplementary Fig. S1). Similarly, the GO cellular terms related to integral component of membrane (G0:0016021) and nucleus (GO:0005634) were found to be enriched in DEGs in response to heat stress (Supplementary Fig. S2).

### Pathway analysis of differentially expressed genes

**Figure 6:**
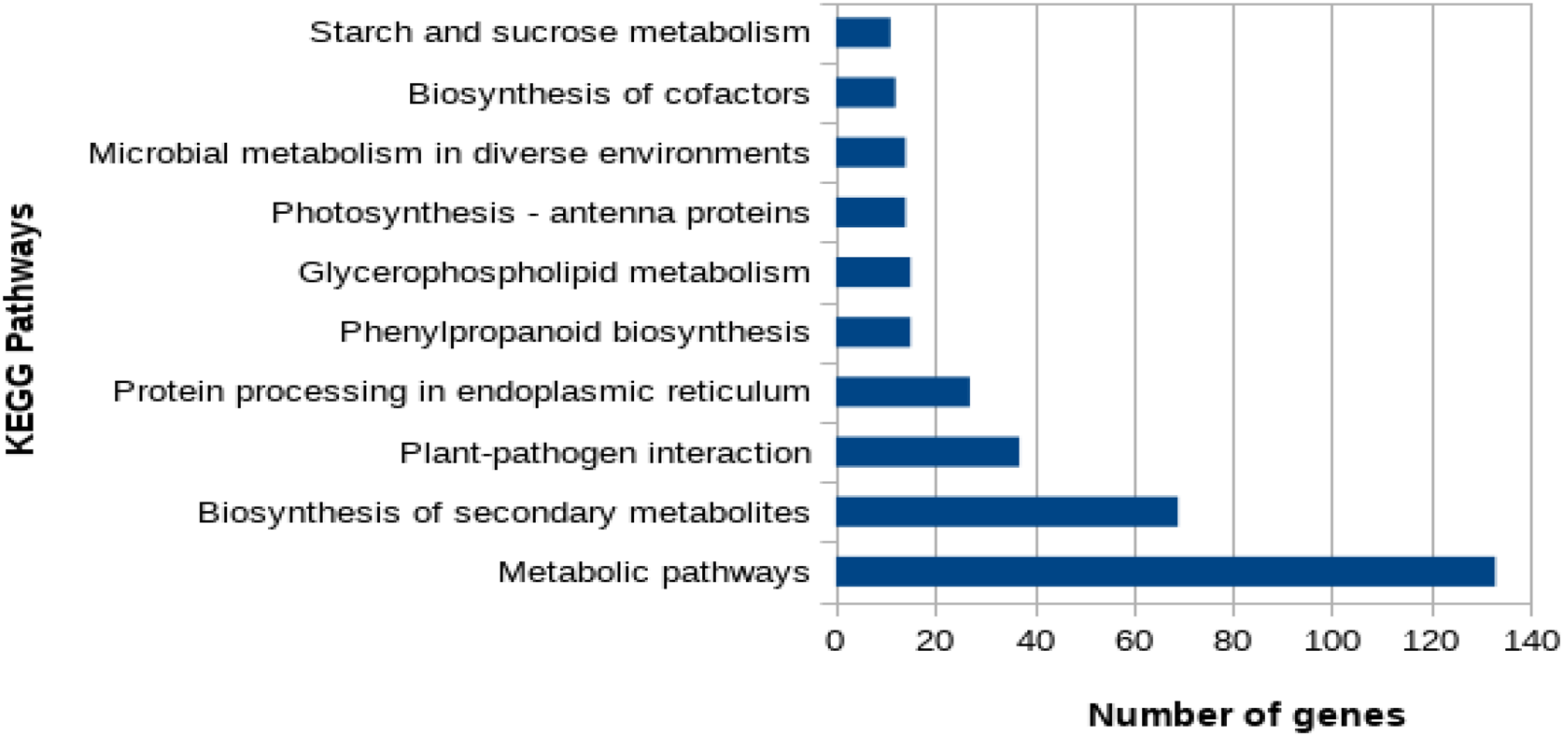
Graphical representation of the top 10 pathways of DEGs during heat stress treatment.

The pathways analysis with KEGG database was performed to determine the pathways in which the DEGs were likely to be associated with. The KEGG analysis results revealed 146 pathways playing roles in different stress tolerance, involving 814 DEGs (Table 2; Fig. 6). Out of this, 133 DEGs were involved in metabolic pathways, 69 DEGs in secondary metabolites biosynthesis pathway, 37 DEGs involved in Plant-pathogen interaction pathway, and 27 DEGS playing roles in protein processing in endoplasmic reticulum.

**Table 2:** List of top 10 pathways of DEGs during heat stress treatment.

S/N	Pathway	Unigene
1	Metabolic pathways	133
2	Biosynthesis of secondary metabolites	69
3	Plant-pathogen interaction	37
4	Protein processing in endoplasmic reticulum	27
5	Phenylpropanoid biosynthesis	15
6	Glycerophospholipid metabolism	15
7	Photosynthesis—antenna proteins	14
8	Microbial metabolism in diverse environments	14
9	Biosynthesis of cofactors	12
10	Starch and sucrose metabolism	11

The analysis further revealed that heat stress treatment of wheat genotype Raj 3765 at 42 °C for 6 h also specifically affected pathways including phenylpropanoid biosynthesis, Glycerophospholipid metabolism, Photosynthesis, Microbial metabolism in diverse environments, Biosynthesis of cofactors, etc.

### Identification of transcription factors, and distribution and frequency of SSRs in the *Triticum aestivum* L. transcriptome

**Figure 7:**
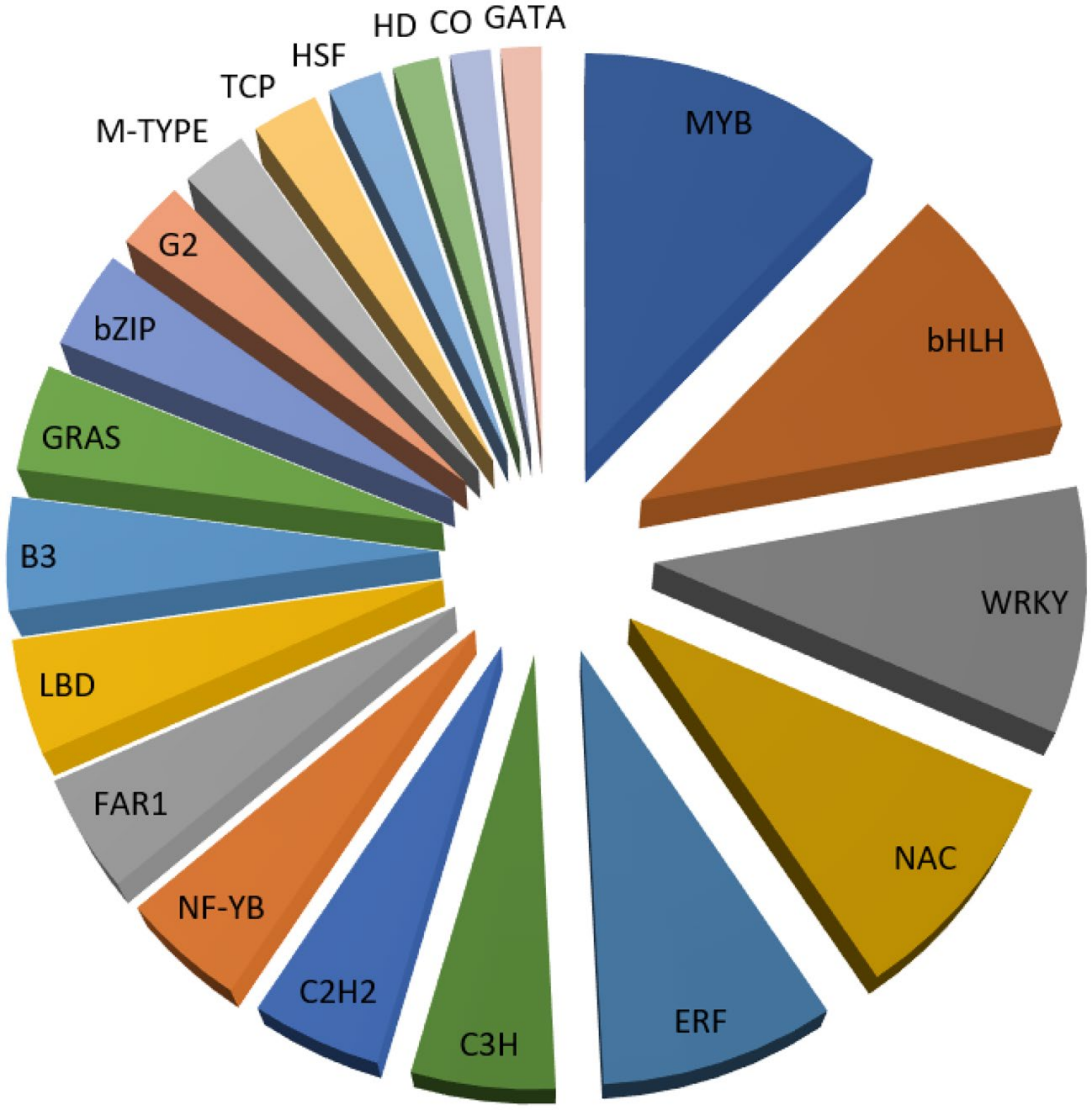
The first twenty transcription factors (TFs) identified in the DEGs.

Transcription factors play very significant regulatory roles in gene expression in relation to heat stress. We identified various transcription factors (TFs) in the DEGs. A total of 1909 transcripts encoding different TFs were identified. Among the differentially expressed TFs, MYB, bHLH, WRKY, NAC, ERF, C3H, and C2H2 were most prevalent (Fig. 7; Table 3).

**Table 3:** List of the first twenty TFs identified in the DEGs.

TF categories	Total
MYB	199
bHLH	168
WRKY	159
NAC	151
ERF	142
C3H	87
C2H2	79
NF-YB	76
FAR1	75
LBD	74
B3	73
GRAS	69
bZIP	64
G2	45
M-TYPE	43
TCP	43
HSF	35
HD	30
CO	26
GATA	26

A total of 612 SSRs were identified from the 7990 sequences examined. Out of the 612 SSRs identified, 542 contain sequences. The number of sequences containing more than one SSR was 62 and 35 compound SSRs were observed (Table 4).

**Table 4:** Distribution and frequency of SSRs in the *Triticum aestivum* L. transcriptome.

Total number of sequences examined	7990
Total size of examined sequences (bp)	6,823,736
Total number of identified SSRs	612
Number of SSR containing sequences	542
Number of sequences containing more than 1 SSR	62
Number of SSRs present in compound formation	35

### Expression of heat shock proteins (HSPs) in response to heat stress

The transcriptional response of wheat genotype Raj 3765 to heat stress showed differential expression log2 |FC| ≥ 2 of a large number of transcripts, including HSPs, SHSP domain-containing proteins, HSF domain-containing proteins and heat stress transcription factors (supplementary Table S1). A total of 40 transcripts encoding heat stress associated proteins and transcription factors were differentially expressed, among which 31 genes were upregulated and 9 were down regulated as compared to control (supplementary Table S1).

### Validation of candidate DEGs by real time-quantitative PCR

**Figure 8:**
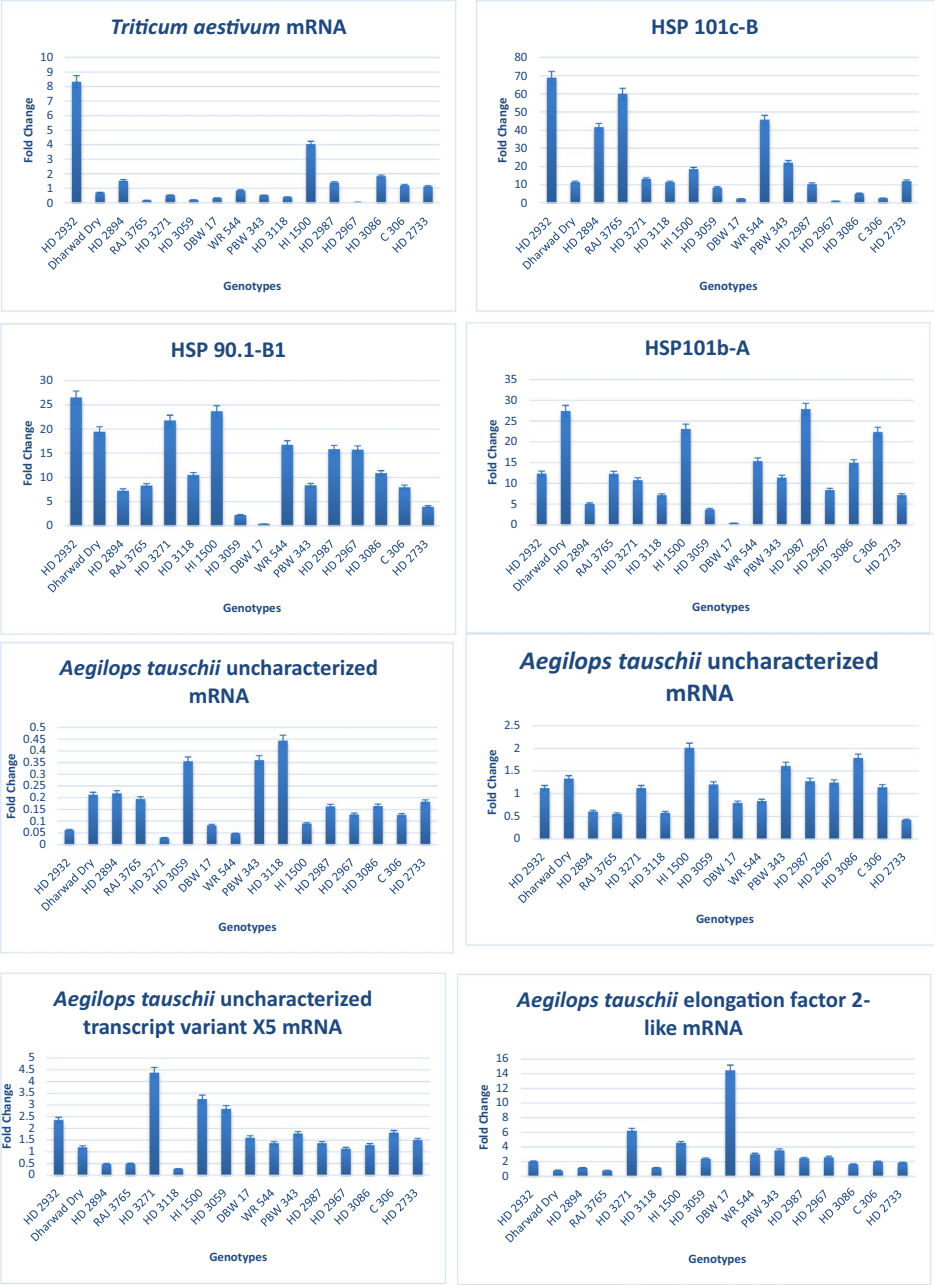
Validation and expression of eight selected genes detected by real time-quantitative PCR (RT-qPCR). Gene expression levels were normalized to the internal control *TaActin*.

To further ensure that the results from the RNA-seq data were reliable, RT-qPCR was carried out to determine the level of expression of eight selected DEGs, including four (4) upregulated and four (4) down regulated genes. Real time-quantitative PCR results showed positive correlation with the transcript abundance changes from RNA-seq data (Fig. 8). Differences in these genes' expression levels between RT-qPCR and RNA-seq could have occurred from errors in repeated trials or varying sensitivities and corresponding algorithms between the two analysis methods.

## Discussion

High temperature is one of the major climatic conditions which adversely affects both plant growth and development, resulting into drastic loss of crop yield^20,21^. High temperature-induced heat stress can also inhibit photosynthesis and increase the rate of photorespiration and transpiration of the plants^20,22^. The advance in biotechnology has allowed rapid genetic gains in plants, but identification of the critical genes for heat stress tolerance and deciphering the heat stress tolerance mechanism, thereof remains a challenge.

The volcano plot revealed that, there were more up-regulated transcripts than the down-regulated ones. This implies that more genes were positively expressed in the flag leaf of Raj 3765 in response to heat stress.

KEGG pathway enrichment analysis showed significant enrichment in metabolic pathways and secondary metabolites biosynthesis pathways. This is probably an indication that the level of expression of genes involved in these pathways changed significantly in response to the temperature-induced heat stress. High temperature-induced heat stress has the potential of causing disturbances in the metabolic pathways in cells of plants. This results in the increase or decrease in the amount of some metabolites and proteins such as osmo-protectants and anti-oxidative enzymes^23,24^.

A number of studies involving transcriptome profiling of plants under heat stress indicated that metabolic pathways and secondary metabolite biosynthetic pathways were the most significantly enriched pathways of the identified DEGs in *Brassica napus* L.^25^, maize^12,26,27^, perennial grass^28^ and wheat^29^.

Transcription Factor (TF) identification revealed that 1909 genes were TFs. Out of this, a total of 199 transcripts were found to be MYB related TFs. MYB-related transcription factors are vital telomere-binding proteins that help to maintain the integrity of the chromosomal structure and also to regulate gene transcription. This family of TFs are chiefly involved in protein–protein interaction, binding of DNA and protein regulation^30^. Various studies in different plants have established the presence and role of MYB TFs in regulating plants response to biotic and abiotic stress. In wheat, *TaMYB80* was found to be effective for heat and drought stress tolerance in transgenic Arabidopsis^31^. Overexpression of *OsMYB1* gene in rice has the potential of conferring tolerance to both heat and salinity stresses^32^, while maize *OsMYB55* enhanced drought and heat stress tolerance^33^. The presence of the high number of *MYB*-related transcription factors in the present study could therefore be playing roles in the thermotolerance in wheat genotype Raj 3765.

A total of 168 transcription factors were considered *bHLH*, 159 as *WRKY*, 151 as NAC related, and 142 as ERF. Studies revealed that constitutively expressing *TaWRKY1* and *TaWRKY33* enhanced thermotolerance in Arabidopsis^34^. Similarly, expressing *TaWRKY70* in wheat improved upon the thermotolerance^35^. Furthermore, when *TaWRKY008, TaWRKY122* and *TaWRKY45* were overexpressed in wheat, there was an increase in the level of tolerance to heat stress^36^. Since many studies revealed the function of *WRKY* transcription factors in thermotolerance, we believe that their presence in the present study could be contributing to the level of thermotolerance.

In the same vein, the presence of NAC family of TFs in the heat stress-responsive transcriptome data of wheat genotype Raj 3765 could be an indication of the tolerance level of the genotype to heats stress since NAC has been implicated in thermo-tolerance in many studies. NAC is one of the most essential and biggest plant-specific stress-responsive TFs^37^. NAC genes have been reported to function in heat stress tolerance. For example, *NTL1* and *NTL11* genes were found to be overexpressed of were observed Arabidopsis after subjecting them to heat stress^38^.

It was revealed that overexpressing *TaNAC2L* in Arabidopsis led to an improvement in the acquired thermotolerance which also activated the expression of other heat-related genes^39^. This is probably a confirmation that the presence of NAC TFs in the transcriptome data of wheat genotype Raj 3765 could be playing a direct role in its thermo-tolerance, or may be involved in regulating the expression of other stress-related genes in order to confer thermotolerance in the genotype.

ERF (Ethylene Responsive Factors) TFs play major functions in conferring tolerance to many abiotic stresses. DREBs proteins are the most characterized ERF in response to abiotic stress. Overexpressing *TaDREB3-A1* gene led to an increase in the level of tolerance against heat, drought and salt stresses in Arabidopsis^40^. The presence of bHLH TFs could also be playing roles in enhancing the heat stress tolerance level of the wheat genotype Raj 3765. A number of the *bHLH* genes identified in various major crops such as rice, wheat (*Triticum aestivum* L.), and maize were determined to function in the plants’ responses to abiotic stresses^41^. Arabidopsis *bHLH112* was determined to be functional in regulating the expression of genes involved in abiotic stress tolerance^14^.

A number of studies have revealed the roles of *HSPs* in conferring thermotolerance in plants. Heat shock proteins (*HSPs*) are known to belong to a large group of molecular chaperones, which function in protein folding and protein assembly; as well as translocation and degradation in order to protect plants from abiotic stress-related damages^42^. Over-expression of wheat *sHSP* gene, *TaHSP26*, led to enhanced tolerance to heat stress in transgenic Arabidopsis^43^. Similarly, when *OsHSP18.6* was overexpressed in rice, it increased the thermotolerance in the rice plants by inhibiting the damaging effects of ROS^44^. Various studies indicate that *Hsfs* are functional in the response of plants to heat stress. For example, *A. thaliana HsfA2*-mutant plants were reported to be more sensitive to heat stress at 37 °C when compared to the its wild-type plants^45^. The expression of *OsHsfA2e* improved upon the level of thermotolerance and salt tolerance in *A. thaliana*^46^.

In this study, few sHSPs genes were found to be significantly up-regulated in the transcriptome data (Fig. 7; supplementary Fig. S2; supplementary Table S1). Three of them (*HSP 90.1-B1, HSP 101b-A,* and *HSP 101c-B*) were selected for validation using RT-qPCR. All of these showed consistent up-regulation in the RT-qPCR, which showed that they could actually be playing vital role in the thermotolerance of the wheat plant.

Our RT-qPCR analysis of all the selected genes revealed that the expression pattern of the validated genes basically agrees with the RNA-seq results. We speculate that the up-regulated genes may be involved in important roles in thermotolerance in wheat.

SSRs have been widely used in genetic diversity analysis, QTL mapping, genome-wide association studies, and marker-assisted breeding^47,48^. The presence of SSRs in intergenic regions of the genome, such as transcriptome sequences, can help in the development of SSR markers from these regions, which can act as functional genetic markers to be widely used for marker assisted breeding and genomic selection^49,50^.

A number of studies^51,52^ used SSR markers to characterize heat stress tolerant wheat germplasm. We believe that the presence of the SSRs in our transcriptome data give an indication of their possible role in heat stress response in wheat genotype Raj 3765. We suggest that further studies should be carried out to validate this SSRs for possible development of SSR markers that can be useful in marker-assisted breeding of heat stress tolerant wheat genotypes.

## Conclusion

The results of this study provide a basis for further research into the functions of genes and the mechanism of thermotolerance in wheat.

We therefore believe that these results could help to enhance our understanding of the mechanism underpinning heat stress tolerance in wheat. The identified genes could also be used as potential candidate genes for developing heat-tolerant wheat cultivars.

## Supplementary Information

Supplementary Figure S1.

Supplementary Figure S2.

Supplementary Table S1.

## Data Availability

All data relevant to the study are included in the article or uploaded as Supplementary Information. In addition, the datasets used and/or analysed during the current study are available in the Sequence Read Archive (SRA) repository with SRA IDs; SRR16347581, and SRR16347579.
