# Supplementary material for: Transcriptome profiling reveals the genes and pathways involved in thermo-tolerance in wheat (Triticum aestivum L.) genotype Raj 3765
Source: Sci Rep. 2022 Sep 1;12:14831. doi: 10.1038/s41598-022-18625-7 (PMC9437100; doi:10.1038/s41598-022-18625-7)
Supplement: Supplementary file 1 — Supplementary Figure S1. [file 41598_2022_18625_MOESM1_ESM.pdf]

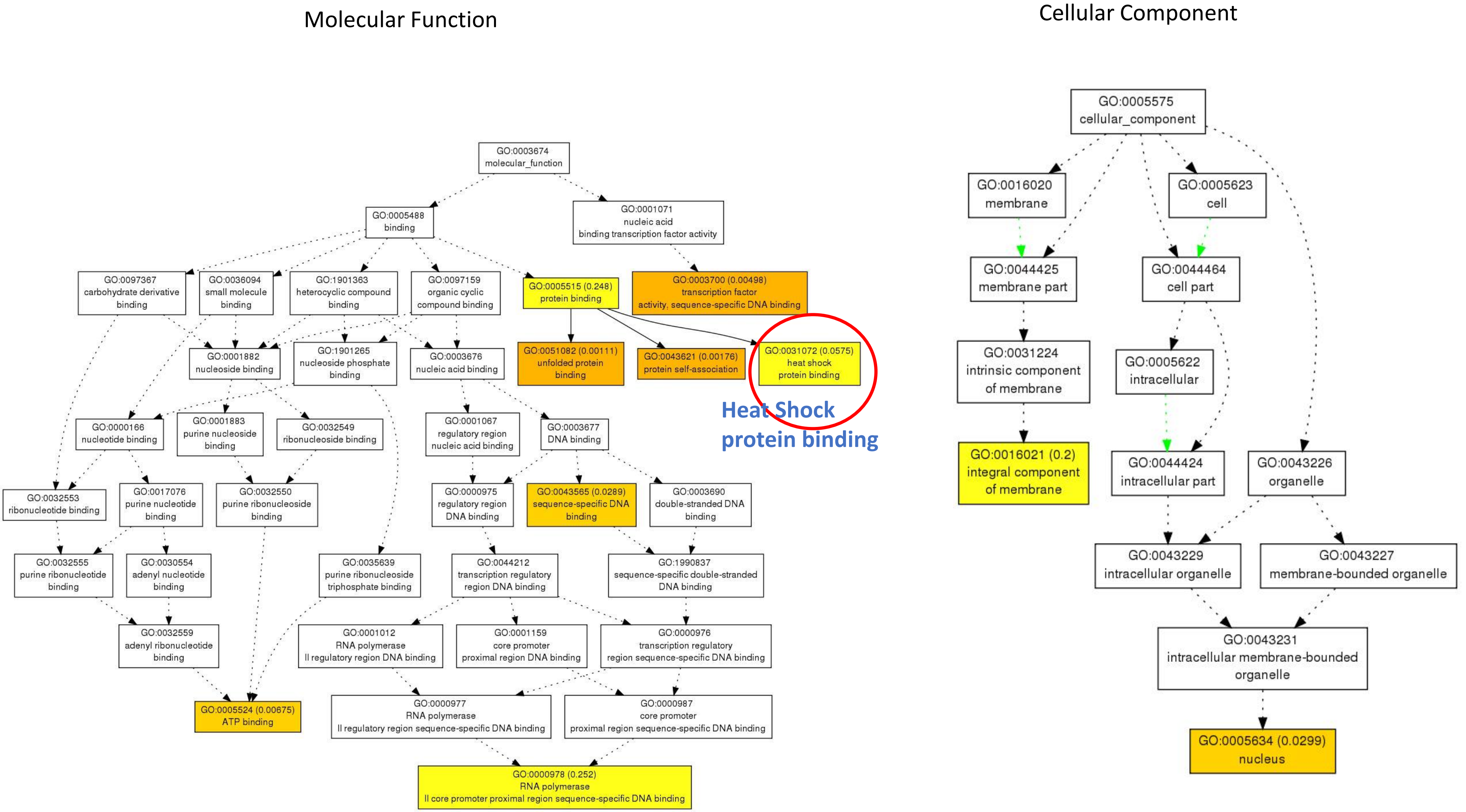

Fig S1: Gene Ontology Enrichment Analysis: Molecular function and cellular component are enriched after heat stress. Biological process are not significantly enriched in GO terms. Boxes in the graph represent GO terms with their GO ID, term definition and statistical information. Significant GO terms (p≤0.05) are marked with color and non-signifcant GO terms are shown in white boxes. The degree of colour saturation of a box is positively correlated to the enrichment level of the term. Solid, dashed, and dotted lines represent two, one and zero enriched terms at both ends connected by the line, respectively. The rank direction of the graph is set to from top to bottom.
