# Supplementary material for: Transcriptome profiling reveals the genes and pathways involved in thermo-tolerance in wheat (Triticum aestivum L.) genotype Raj 3765
Source: Sci Rep. 2022 Sep 1;12:14831. doi: 10.1038/s41598-022-18625-7 (PMC9437100; doi:10.1038/s41598-022-18625-7)
Supplement: Supplementary file 2 — Supplementary Figure S2. [file 41598_2022_18625_MOESM2_ESM.pdf]

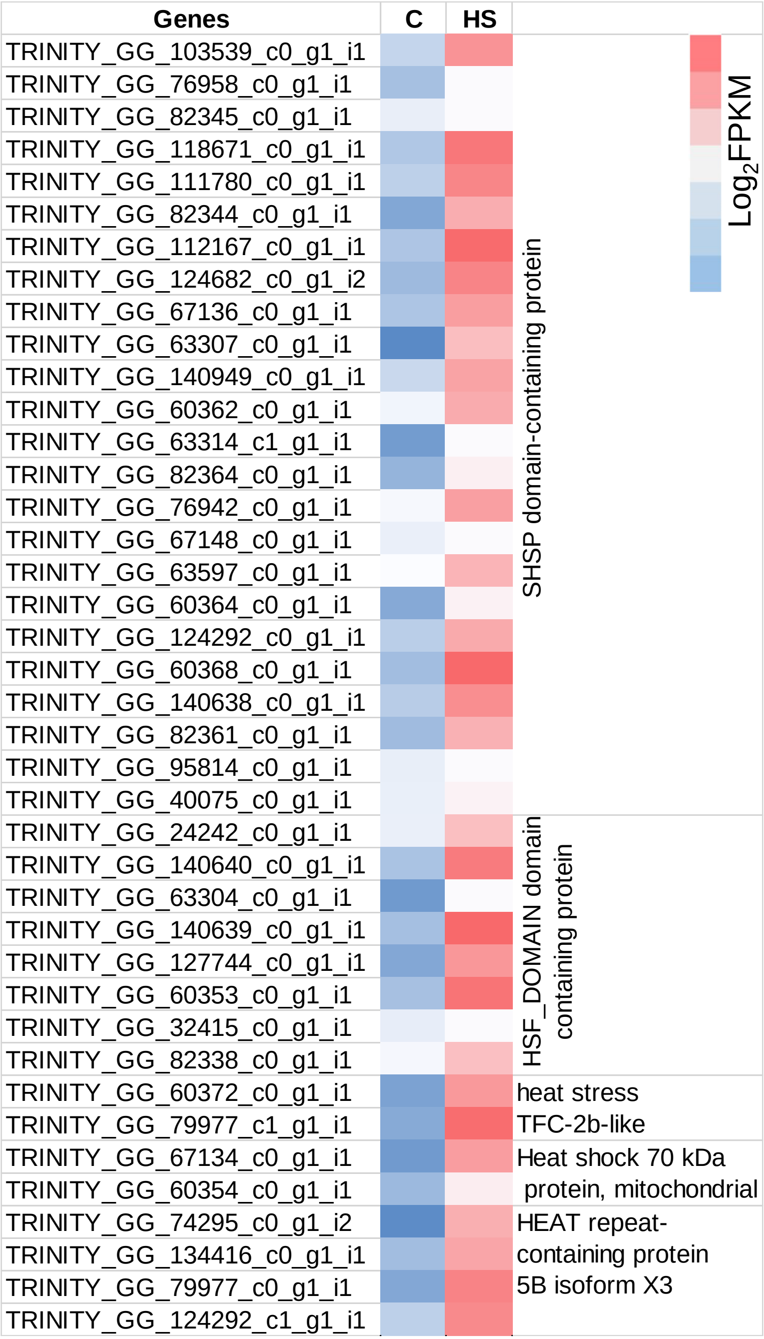

Fig S2: Heatmap of expression levels of differentially expressed heat stress related protein gene: Heatmap of expression levels (in the form of FPKM values, FPKM is the fragments per kilobase per million fragments mapped) of differentially expressed heat stress related protein gene. Red colour is for upregulation and blue colour for downregulation. C represents control and HS is heat stress treatment.
